# The Thermometer in Obstetrics and Gynæcology

**Published:** 1891-06

**Authors:** 


*The Thermometer in Obstetrics and Gynæcology*. By A. D. LEITH NAPIER, M.D. London: H. K. LEWIS. 1890.

This pamphlet consists of two lectures delivered at the Midwives' Institute. They must have been uncommonly dreary for the listeners, for they contain little else than the enumeration of various readings of the thermometer after childbirth and after the graver gynæcological operations.

The thermometer is of course an indispensable aid in diagnosis, but it is easily possible to overrate its value. Too often a high temperature is looked upon as a disease which has to be attacked by a liberal exhibition of so-called anti-pyretics, when the more scientific and satisfactory course is to seek out the cause and remove it. One free saline purge will often do more than numerous doses of recently-introduced drugs.

However, Dr. Leith Napier's lectures, founded upon an extensive personal experience, were worth printing; although we fail to see, when addressed to nurses, the desirability of the latter portion dealing with treatment by medicines.

